# Evaluation of Nurse-Implemented Self-Management Interventions for Patients with Chronic Inflammatory Arthritis in Bulgaria

**DOI:** 10.3390/jcm14144854

**Published:** 2025-07-09

**Authors:** Stefka Stoilova, Mariela Geneva-Popova, Stanislava Popova-Belova

**Affiliations:** 1 Department of Health Care Management, Faculty of Public Health, Medical University of Plovdiv, 4002 Plovdiv, Bulgaria; 2 Department of Propaedeutics of Internal Diseases, Faculty of Medicine, Medical University of Plovdiv, 4002 Plovdiv, Bulgaria; mariela.geneva@mu-plovdiv.bg (M.G.-P.); stanislava.popova@mu-plovdiv.bg (S.P.-B.)

**Keywords:** biologic disease-modifying antirheumatic drugs (bDMARDs), chronic inflammatory arthritis (CIA), nurses’ role, self-management, self-injection, healthy lifestyle, psychological support

## Abstract

**Objective:** To evaluate the role of nurses in self-management interventions for chronic inflammatory arthritis (CIA). Key areas of interest included the following: (1) providing education on self-injection techniques for biologic disease-modifying antirheumatic drugs (bDMARDs), (2) promoting healthy lifestyles, and (3) supporting mental health. Patients’ satisfaction with the care received was also examined. **Methods:** A cross-sectional study involving CIA patients, rheumatologists, and nurses was conducted. Participants assessed nurses’ competence in areas such as training for bDMARD self-injection, lifestyle guidance, and emotional support. Satisfaction scores and preferences in managing side effects were also analyzed. **Results:** The participants expressed high confidence in the nurses’ ability to support CIA self-management. The patients rated the nurses significantly higher than doctors in training for self-injection (*p* = 0.002) and offering guidance on nutrition and healthy habits (*p* = 0.002). Although it was not a statistically significant difference, the patients also showed stronger trust in the nurses’ ability to provide psychological and emotional support. Most patients (93.0%) would contact a rheumatologist in case of side effects; 35.5% would seek a nurse. The patients attended by both a doctor and nurse reported significantly higher satisfaction compared to those seen only by a rheumatologist (*p* < 0.001). Both the doctors and nurses acknowledged the importance of the nurse–patient relationship for effective care (*p* = 0.527). **Conclusions:** The findings highlight the critical role of nurses in patient education—particularly in training for self-injection and promoting a healthy lifestyle and mental well-being. Their involvement is strongly associated with higher patient satisfaction and contributes significantly to the overall effectiveness of care in CIA management.

## 1. Introduction

Nurses play essential roles in the field of rheumatology. Current recommendations from the European Alliance of Rheumatology Associations (EULAR) advocate for the active involvement of nurses in the comprehensive management of patients with chronic inflammatory arthritis (CIA) [1]. These recommendations emphasize the importance of providing timely access to nursing care, ensuring patient satisfaction, managing the disease effectively, offering psychosocial support, promoting self-management, and delivering education tailored to patient needs. To prevent the progression of CIA, the therapeutic management of these patients should be individualized and may encompass both pharmacological and non-pharmacological interventions. Non-pharmacological interventions can include exercise, education, rehabilitation, disease information, and counseling, as well as psychological support and education [2].

EULAR has detailed the methods of delivery and the theoretical frameworks for educating patients with inflammatory arthritis (IA), recognizing that patient education is a crucial component of self-management interventions throughout the progression of the disease. A strong understanding of the disease, medications, treatment objectives, and prognosis can enhance patients’ adherence to treatment and lead to better outcomes [3].

Biologic disease-modifying antirheumatic drugs (bDMARDs) are indicated for treating patients with CIA, including rheumatoid arthritis (RA), psoriatic arthritis (PsA), and ankylosing spondylitis (AS). The administration of bDMARDs occurs through subcutaneous injections or intravenous infusions and requires ongoing monitoring and management of the treatment process. Given the long-term nature of this therapy, patients are encouraged to learn self-injection techniques. It is believed that self-injection may improve treatment adherence compared to receiving injections from family members or in clinical settings [4].

Optimal management of CIA is achieved through a multidisciplinary team (MDT) that includes various medical professionals, among them nurses [5]. One of the responsibilities of nurses in the MDT is to educate patients in self-injection techniques for subcutaneously administered medications, monitor safety, and coordinate treatment [6]. There is debate about how to train patients to self-inject subcutaneous bDMARDs, as there is a lack of clear guidance about which healthcare professionals should conduct this training. Pharmaceutical companies that manufacture bDMARDs do not provide training programs but offer detailed information on use and visual demonstrations on their websites [7]. According to the literature, nurses are currently training patients in self-injection techniques for other conditions, including diabetes, multiple sclerosis, and osteoporosis [4,8,9,10]. Educational sessions led by nurses emphasize the importance of delivering personalized care tailored to each patient’s unique needs [11]. These sessions aim to ensure patient safety and monitor adherence to prescribed treatments.

To effectively manage their condition, patients with CIA must be recognized as partners, placed at the core of the patient–professional relationship within the biomedical care process [12]. It is beneficial for patients to receive education on proper injection techniques from healthcare professionals, such as physicians or nurses, when starting their first subcutaneous bDMARD [5,13]. According to Schiff M et al., healthcare professionals (including physicians and nurses) can assist CIA patients by teaching them self-injection techniques for bDMARDs, enabling them to develop their own self-injection skills [14]. Educating patients to self-inject offers numerous advantages, such as improved adherence to treatment [15], enhanced treatment outcomes, greater satisfaction, and, most importantly, the empowerment and autonomy of individuals who are able to take control of their condition.

The effects of self-injection training extend to the healthcare system by decreasing the number of hospitalizations and outpatient visits for routine injections, which in turn leads to reduced healthcare costs [16]. Patients are empowered to take responsibility for their treatment, fostering independence from health professionals. This independence allows for greater flexibility in the timing and location of injections, enabling patients to self-inject at home, at work, or even while traveling [17,18,19]. Therefore, patient education, training, and support are critical factors that influence the success or failure of self-injection [14].

The EULAR recommendations for CIA patient education specify when and by whom patient education should be offered [20]. Part of patient education (PE) is inclusion in self-management programs [21]. Self-management interventions and their role in relation to medical treatment can vary. Some of them, with the active participation of nurses, are related to promoting physical activity, advice on lifestyle changes and adopting healthy habits, providing mental health support, and undertaking appropriate interventions when needed [22]. According to the recommendations, healthcare professionals should be aware of the benefits of physical activity, which should be considered an integral part of standard patient care and actively encouraged but also tailored to the individual needs and capabilities of the patient throughout the course of the disease.

The implementation of educational activities focused on lifestyle changes for patients and the promotion of healthy habits presents challenges for healthcare professionals. In response, EULAR developed recommendations in 2021 regarding lifestyle and work participation aimed at preventing the progression of rheumatic and musculoskeletal diseases (RMDs) [23]. These lifestyle recommendations identify six key factors—exercise, diet, weight control, alcohol consumption, smoking, and work participation—across seven types of RMDs: osteoarthritis, rheumatoid arthritis, axial spondyloarthritis, psoriatic arthritis, systemic lupus erythematosus, systemic sclerosis, and gout. A report by Wojeck et al. [24] indicated that nurse-led interventions for patients with autoimmune rheumatic diseases enhanced both the quality of life and mental health of the patients involved [25]. Other research has explored the needs and expectations of CIA patients concerning the care they receive, highlighting various challenges these patients face and the emotional strain of living with a chronic illness [26].

The current study aimed to evaluate the role of nurses in self-management interventions for chronic inflammatory arthritis (CIA) by examining the perspectives of CIA patients, rheumatology specialists, and nurses on several key issues: (1) providing education on self-injection techniques for biologic disease-modifying antirheumatic drugs (bDMARDs); (2) promoting healthy lifestyles; and (3) supporting mental health. Additionally, the study explored patients’ preferences for healthcare professionals with whom to discuss adverse reactions and assessed their satisfaction with the care they received.

## 2. Materials and Methods

The present study utilized a cross-sectional survey design that included three groups of respondents: (1) patients with chronic inflammatory arthritis (CIA), such as rheumatoid arthritis (RA), psoriatic arthritis (PsA), and ankylosing spondylitis (AS); (2) rheumatology specialists; and (3) nurses employed in rheumatology clinics in Plovdiv, Bulgaria.

The study protocol received approval from the scientific ethics board at Medical University—Plovdiv, Bulgaria (approval code No. P-17188, approval date: July 2024). All procedures were conducted in compliance with the World Medical Association Declaration of Helsinki (1964) and its 2000 modification (Edinburgh). Written informed consent was obtained from the participants regarding the publication of data in scholarly journals.

The survey questions, in addition to collecting demographic data, gathered responses about nurses’ roles in helping patients manage their disease. This included assisting patients in learning to administer self-injections of bMARDs, coping with potential side effects, engaging in physical activities, adopting healthy nutrition habits, controlling smoking and alcohol consumption, and maintaining an adequate psychological and emotional state. The questions were presented on a five-dimensional Likert scale: 1 = “no,” 2 = “rather no,” 3 = “I can’t judge,” 4 = “rather yes,” and 5 = “yes.” Patients also expressed their degree of satisfaction with the care provided and assessed their health status using a visual analog scale (VAS) from 0 (worst health imaginable) to 100 (best health imaginable).

A core concept in the survey is related to nurses’ competence in fostering patient independent skills in self-injection of bMARDs, healthy lifestyle, and emotional stability. As part of their medical education in Bulgaria, nurses go through four years of specialized training and get a bachelor’s degree that meets the European Union’s criteria for the profession. The Bulgarian Association of Health Professionals in Nursing (BAHPN) has been an official member of the International Council of Nurses (ICN) since August 2008. On 16 January 2009, BAHPN also joined the European Federation of Nurses Associations (EFN). Since 2006, BAHPN has also been a member of the European Forum of National Nursing and Midwifery Associations (EFNNMA) and the World Health Organization (WHO) [27]. In the context of this research, a nurse in Bulgaria who specializes in chronic inflammatory arthritis should be able to provide holistic care, which includes the regular nurse’s duties, as well as coaching patients to make adjustments to their lives for better physical and emotional well-being.

Regarding emotional support, the interventions implemented by the nurses are limited to maintaining effective communication with patients, having therapeutic conversations while actively listening, and building a trusting relationship with each other. As part of continuing education, most nurses, including those participating in the present study, undertake training in “Neuropsychology, Psychopathology, and Therapy” with a corresponding certificate [28].

### Statistical Methods

The statistical software IBM SPSS Statistics for Windows, Version 28.0 (Armonk, NY, USA: IBM Corp.), was utilized for data analysis. Continuous variables were evaluated for normality using the Shapiro–Wilk test. When normality was confirmed, the results were reported as mean values along with standard deviations (SDs). If normality was not observed, the results were summarized with the median values and interquartile ranges (IQRs). Between-group comparisons were carried out through the Mann–Whitney U test. Ordinal and categorical variables were presented as counts and percentages. To examine dependencies among these variables, Fisher’s exact test was employed for dichotomous variables, while the chi-square test and z-test with Bonferroni corrections were used for pairwise comparisons. All the statistical tests were two-tailed and deemed significant at *p* < 0.05.

## 3. Results

### 3.1. Background Information About the Participants in the Study

A total of 291 participants completed the survey, including 228 (78.40%) CIA patients on injectable bMARD treatment, 29 (10.00%) rheumatology doctors, and 34 (11.70%) nurses working in rheumatology clinics across eight hospitals in Plovdiv, Bulgaria. The work experience of the rheumatology doctors ranged from 4 to 48 years, with an average of 19.50 ± 12.19 years. The nurses had a mean work experience of 23.53 ± 11.90 years, with a range from 1 to 49 years.

**Figure 1:**
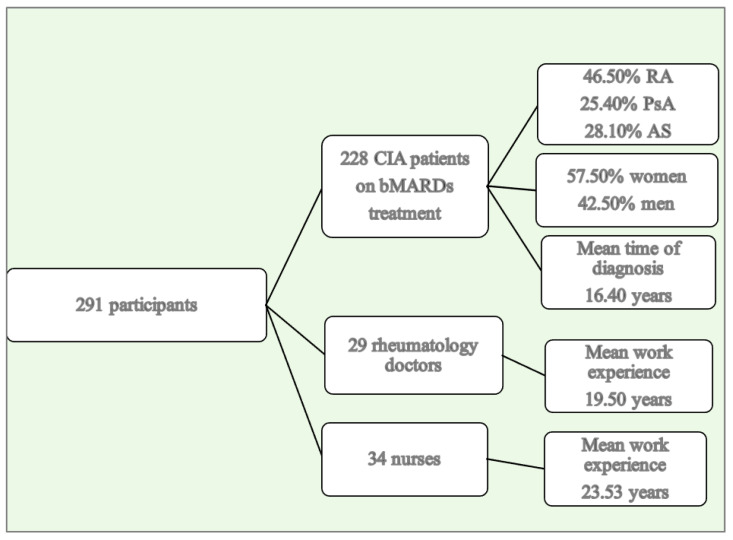
Participants in the study.

The ages of the patients varied from 20 to 80 years, with a mean age of 56 ± 13.37 years. The sex distribution showed that 57.50% (n = 131) of the participants were women, while 42.50% (n = 97) were men. The diagnoses included 106 (46.50%) patients with rheumatoid arthritis (RA), 58 (25.40%) with psoriatic arthritis (PsA), and 64 (28.10%) with ankylosing spondylitis (AS). The time since diagnosis ranged from 4 to 54 years, with a mean of 16.40 ± 9.40 years. All the patients were receiving therapy with bMARDs for a duration ranging from 1 year to 12 years, with a median duration of 6 years (IQR = 5) (Figure 1).

### 3.2. Participants’ Perceptions Regarding the Ability of Nurses to Coach Patients on Self-Injection of bMARDs, Promote Healthy Living, and Support Psychological Well-Being

Out of 228 CIA patients who received injectable bMARDS, 169 (74.10%) learned to self-inject the medication from a nurse, 41 (18.00%) received training from a rheumatology doctor, 15 (7.00%) were trained by a general practitioner, and 2 (0.90%) were taught by others, such as family members or friends.

The majority of respondents in all three groups expressed positive views about the nurses’ competence to train patients in self-injection, with 88.1% of patients, 79.4% of nurses, and 65.5% of doctors answering “Yes”. While only 5.9% of patients and 17.6% of nurses selected “Rather yes”, a significantly higher proportion of doctors (31.0%) chose this more reserved response. Overall, patients were significantly more likely than doctors to respond affirmatively (χ^2^ = 21.18, df = 2, *p* = 0.002). Neutral and negative responses were minimal across all groups.

The participants’ opinions on nurses’ expertise in advising patients with CIA on physical activity varied across the three respondent groups; however, the differences were not significant (χ^2^ = 13.83, df = 2, *p* = 0.086). The majority of patients (68.5%), nurses (55.9%), and doctors (51.7%) responded “Yes”, indicating overall confidence in nurses’ ability to provide physical activity guidance. An additional 16.4% of patients, 26.5% of nurses, and 31.0% of doctors expressed a more reserved response “Rather yes”. Neutral responses were more common among doctors (13.8%) and patients (10.5%) compared to nurses (2.9%). A small percentage of respondents selected negative options (“Rather no” or “No”).

Significant differences were found between the groups regarding perceptions of nurses’ expertise in offering guidance on nutrition, harmful habits, and other health-related issues to patients with IJD (χ^2^ = 24.41, df = 2, *p* = 0.002). The majority of patients (68.0%) and nurses (64.7%) responded “Yes”, compared to 44.8% of doctors, indicating significantly lower confidence among doctors. Furthermore, 48.3% of doctors selected “Rather yes”, a proportion significantly higher than that of nurses (26.5%**)** and patients (16.4%**),** suggesting more reserved support within the physician group. Neutral responses were reported only by patients (11.4%) and one doctor (3.4%)**,** while nurses reported no neutral answers. Negative responses (“Rather no” and “No”) were infrequent across all the groups.

The majority of the participants expressed strong confidence in nurses’ abilities to provide psychological and emotional support to patients with CIA undergoing treatment with bMARDs. Although the differences were not statistically significant (χ^2^ = 12.71, df = 2, *p* = 0.122), the highest proportion of unequivocal affirmative responses, “Yes”, came from patients (87.7%). On the other hand, nurses (67.7%) and doctors (65.5%) had similar rates of affirmative responses. Additionally, 11.0% of patients, 20.6% of nurses, and 29.7% of doctors provided a more cautious response of “Rather yes”. Neutral responses were more prevalent among doctors (13.8%) compared to patients (5.0%) and nurses (5.9%). A small percentage of both patients and nurses chose negative responses (“Rather no” or “No”), while none of the doctors selected negative options (Table 1).

### 3.3. Recognition and Management of Adverse Reactions (AEs)

Conducting biological therapy involves monitoring patient safety and managing adverse reactions (AEs). Patients have the opportunity to contact various members of the treatment team as needed. Among the 228 CIA patients, 209 (91.60%) reported being informed about the potential side effects of the treatment, while 14 (6.10%) responded, “Rather yes”. Only one patient (0.40%) gave a neutral response, two (0.90%) indicated “Rather no,” and another two (0.90%) answered “No”.

**Figure 2:**
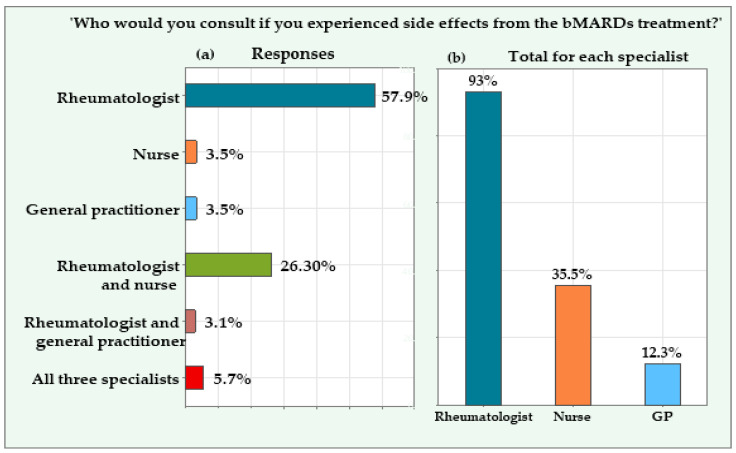
Panel (**a**) shows the distribution of patients’ responses about whom they would consult if they experienced side effects from bMARD treatment. Panel (**b**) presents patients’ overall preferences for each specialist.

If patients experienced treatment side effects, 148 (64.90%) would reach out to one of the three specialists: 132 (57.90%) would see a rheumatology doctor, 8 (3.50%) would consult a rheumatology nurse, and 8 (3.50%) would contact a general practitioner (GP). The remaining 80 (35.10%) patients would contact more than one specialist: 13 (5.70%) would seek help from all three professionals—a rheumatology doctor, a rheumatology nurse, and their GP. Sixty (26.30%) patients would contact both a doctor and a nurse, while seven (3.10%) would consult a doctor rheumatologist and a GP (Figure 2a).

In total, 212 patients (93.00%) would consult a rheumatology doctor, 81 patients (35.50%) would seek help from a nurse, and 28 patients (12.30%) would contact their GP for advice. The total exceeds 100% because, as previously mentioned, 80 patients provided multiple responses (Figure 2b).

### 3.4. Patients’ Satisfaction with the Care Administered by the Attending Staff

According to the medical records, 170 patients (74.60%) were attended by a rheumatology doctor along with other staff members, while 58 patients (25.40%) received care solely from a rheumatology doctor. Among the 170 patients who were attended by a healthcare team including a rheumatology doctor and other staff, 168 were assisted by a nurse, and 2 received care from a doctor’s assistant.

For the next analysis, which assessed the level of satisfaction with the care received from the healthcare staff, we categorized the patients into two groups: (1) patients who were attended by a team including a rheumatology doctor along with a nurse, and (2) patients who were attended by a rheumatology doctor alone. The patients were asked to evaluate the quality of the received care on a scale of 0 to 100, where 0 = not pleased at all and 100 = very pleased.

**Figure 3:**
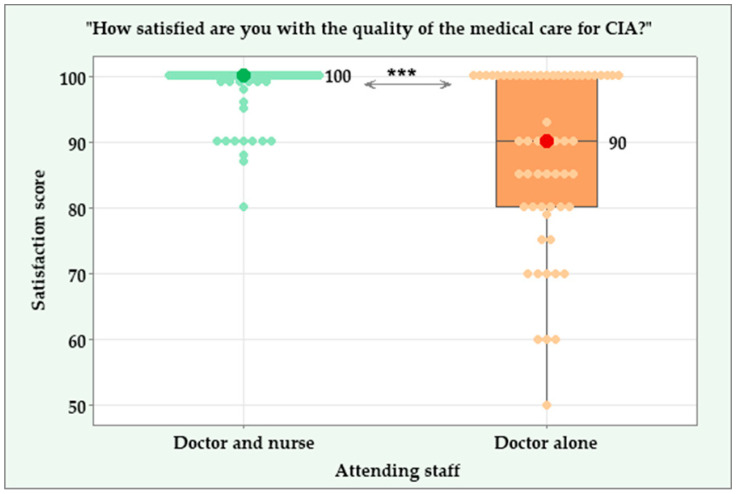
Patients’ satisfaction with the care administered by the attending staff. ***—Significant difference at *p* < 0.001.

The patients who were attended by a rheumatology doctor along with a nurse had a perfect median score of 100 (IQR = 0.00) versus a median score of 90 (IQR = 20) in the group who were attended solely by a rheumatology doctor, with a significant difference (Mann–Whitney U = 7432.00, standardized test statistic = 7.851, *p* < 0.001). The box plots in Figure 3 show a narrow range in the individual scores of the patients attended both by a rheumatology doctor and a nurse, with a minimum score of 80, a maximum score of 100, and an interquartile range of 0. In contrast, the individual satisfaction scores in the group attended solely by a rheumatology doctor vary from 50 to 100, with an interquartile range of 20.

### 3.5. Doctors’ and Nurses’ Opinions on the Role of Nurses in Nurse–Patient and Doctor–Patient Relationships for the Effectiveness of the Treatment

Both doctors and nurses recognized the importance of the nurse–patient relationship for effective treatment. The median score in both groups was 3 (indicating “rather yes”), with an interquartile range (IQR) of 2 and no significant difference (*p* = 0.527).

**Figure 4:**
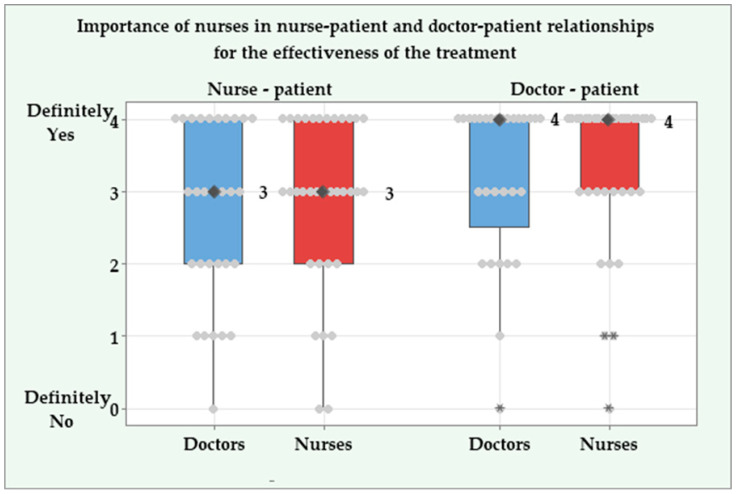
Doctors’ and nurses’ opinions on the role of nurses in nurse–patient and doctor–patient relationships for the effectiveness of the treatment.

Nurses’ role in maintaining effective doctor–patient relationships received an even higher rating, with a median score of 4 in both groups (IQR = 1.5 for doctors and IQR = 1 for nurses) and very few negative responses (*p* = 0.711). The significance of nurses in fostering relationships between nurses and patients, as well as between doctors and patients, is illustrated by the box plots in Figure 4.

## 4. Discussion

The present study is the first survey-based cross-sectional research in Bulgaria to triangulate the opinions of CIA patients, nurses, and rheumatologists on the role of nurses in promoting self-management skills for patients with chronic inflammatory arthritis.

Patients with rheumatoid arthritis, psoriatic arthritis, and axial spondyloarthritis often experience similar symptoms due to the overlapping clinical features of these conditions. Therefore, their treatment employs a similar therapeutic approach. The contemporary management of CIA with bDMARDs includes the delivery of specialized healthcare, where self-management skills are of crucial importance to patients’ quality of life. Awareness of the disease, effective communication with healthcare professionals, and compliance with treatment protocols may enable patients to manage their condition more effectively and engage actively in their own care.

Our data showed that most patients were trained to self-inject bMARDs by a nurse. Significantly fewer patients received training from a rheumatologist or a general practitioner, and very few by family members or friends. The reasons for the higher proportion of nurses involved in the self-injection training process may be multifaceted; however, our results pinpoint the significant confidence in nurses’ competence expressed by all the participants as a determining factor. The most positive evaluations of nurses’ capabilities came from the direct recipients of care—namely, the patients. Some of our other results further confirm this fact by revealing a high appreciation for strong nurse–patient relationships. Our results support a study by Beauvais et al., which indicates that nurse-led training may be beneficial for patients starting bDMARD therapy for the first time [13]. Additionally, our findings align with a study from Bulgaria, which reported positive patient satisfaction with nurse-led training for self-injection of disease-modifying drugs in multiple sclerosis [29].

Training for the self-injection of biologics involves ensuring patient safety. This responsibility includes educating patients and monitoring them for potential adverse reactions (ADRs). In our study, the majority of patients indicated that they received information regarding the possible side effects of their treatment and the opportunity to consult any member of the attending medical staff.

According to our survey results, rheumatologists received the highest level of trust from the patients in the event of adverse reactions, with the majority of the patients stating they would specifically reach out to a rheumatologist. A rheumatology nurse or a general practitioner would be their other choices. Approximately one-third of the patients expressed a preference to contact all three members of the healthcare team, and a substantial number of the patients indicated they would seek advice from both a doctor and a nurse if needed. The importance of education and safety skills for patients receiving bDMARDs for inflammatory arthritis has been highlighted in the study by Rat et al. [30]. They found that patients who did not attend therapy training sessions and lacked consultation with a nurse had lower safety scores [30].

Physical activity interventions have a proven effect in reducing the overall impact of disease, preventing comorbidities [31], and improving patients’ quality of life [32]. In our study, the majority of respondents from all three groups—patients, nurses, and rheumatologists—expressed positive opinions about the ability of nurses to provide physical activity guidelines, without significant differences among them. However, significant differences were observed among the respondent groups concerning their perceptions of nurses’ expertise in offering lifestyle guidance to patients with CIA. Most patients and nurses expressed confidence in nurses’ competence in providing guidance regarding nutrition, managing unhealthy habits, and fostering healthy behaviors in individuals with CIA. In contrast, rheumatology doctors were more cautious in their assessments, showing significantly less confidence in nurses’ capabilities. This was in line with rheumatology doctors’ overall tendency to regard nurses’ qualifications as less credible than those of the other respondents.

The reasons for the observed discrepancy between the patients’ and nurses’ opinions, on one hand, and the more cautious opinions of physicians, on the other, are likely multifaceted. One contributing factor may be that some rheumatology doctors (25.40%) seemed to act as the sole providers of care for their patients, suggesting a lack of teamwork and an overreliance on their expertise. Another factor could be the insufficient collaboration within multidisciplinary teams that include both rheumatologists and nurses. Instead of working collaboratively to achieve comprehensive, patient-centered care, doctors and nurses may experience tensions due to inadequate information sharing and a lack of appreciation for each other’s distinct roles. In this context, local and national programs designed to enhance team dynamics and collaboration could provide a long-term solution to the skepticism, professional bias, and potential tensions that were discerned in this study. A recent article by Miteva et al. (2025) highlights the necessity for systematic health reforms, ongoing training for medical staff, and improved communication techniques through various programs and methods, including digital tools and telemedicine, to ensure effective management of rheumatoid arthritis (RA) and other inflammatory diseases [33].

The literature lacks consensus on which lifestyle changes can significantly impact the progression of CIA. However, EULAR recommendations highlight the importance of supporting patients in adopting healthy habits. These habits include maintaining a healthy and balanced diet, understanding the benefits of exercise, and receiving encouragement to quit smoking [22]. The findings of our study align with those of Den Hamer-Jordaan et al., who reported that nurses possess the skills necessary to promote healthy eating and manage patients’ eating behaviors [34]. They also corroborate the conclusions of Auyezkhankyzy et al., which claimed that nurses provide guidance on healthy lifestyles through education about nutrition, as well as monitoring and controlling weight and eating habits [25].

The majority of the participants expressed strong confidence in nurses’ abilities to provide psychological and emotional support to CIA patients undergoing treatment with bMARDs. Although the differences were not statistically significant, the highest proportion of unequivocal affirmative responses came from patients, followed by nurses and physicians. A similar trend was observed in a study by Dures et al., which reported that 74% of patients preferred receiving psychological help from nurses, 55% preferred physicians, and 51% preferred general practitioners [35]. Despite patients’ high satisfaction with the emotional support provided by nurses, it should be mentioned that the medical training of nurses in Bulgaria should include a stronger focus on cognitive behavioral therapy skills. Nurses should be equipped with proven intervention skills and strategies that would make them even more effective psychological coaches.

Our study indicated that patients receiving care from a team that included both a rheumatologist and a nurse were more satisfied with their care than those who received care solely from a rheumatologist. These findings align with the existing literature. Previous studies have indicated that the clinical effectiveness and cost-effectiveness of nurse-led care (NLC) are comparable to those of rheumatologist-led care (RLC) for patients with rheumatoid arthritis (RA), with overall satisfaction not being inferior in NLC [36,37].

Our findings highlight the crucial role of nurses in building relationships with both patients and physicians. Both nurses and physicians acknowledge that strong nurse–patient relationships contribute to a supportive environment and promote better health outcomes. Our results align with the existing literature, which indicates that positive nurse–patient relationships enhance quality and healing outcomes [38]. Additionally, effective communication between nurses and patients is vital for advancing patient-centered care [39].

Both rheumatology physicians and nurses acknowledge the crucial role that nurses have in fostering effective communication and collaboration between physicians and patients. This collaboration is vital for achieving successful treatment outcomes. The overall quality of healthcare relies significantly on positive relationships among patients, physicians, and healthcare teams [40], which also underlines the importance of adherence to treatment regimens [41].

Our findings are similar to those reported in Marca-Frances et al. [42], who observed that patients were more likely to seek information from nurses because they spend considerable time with them and thus perceive nurses as the most accessible professionals [43]. Nurses can act as advocates for patients and facilitate communication between patients and other healthcare professionals [44]. Additionally, a study by Wieke et al. highlighted that effective communication between healthcare professionals and patients is key to guaranteeing healthcare quality, patient safety, and overall satisfaction [45].

### Limitations

Being survey-based, the results of this study are subject to the inherent limitations of survey designs. Likert scales can introduce desirability biases, especially in questions related to the quality of service and care. Future research should prioritize the collection of more objective data, including clinical reports and standardized evaluations. Additionally, employing qualitative methods such as interviews or focus groups could offer deeper understanding of the subjective aspects of therapy.

Furthermore, the questionnaire on self-management practices did not cover all aspects of patient self-management. The restricted geographical focus inevitably raises concerns about sample representativeness, while the absence of participation from hospitals across the nation further diminishes the generalizability of the findings to the entire Bulgarian healthcare system. Future research on this issue should apply a multicenter strategy that includes a wide range of areas and healthcare settings throughout Bulgaria to increase the external validity of the findings. Additionally, the interventions provided by nurses may be linked to a particular level of competence that may not be uniform among all health professionals across the nation. Moreover, the analysis of nurse-led interventions was broad and lacked details regarding self-injection training, components of a healthy diet, types of exercise, and the types of psychological support provided.

## 5. Conclusions

The findings of our study point out the importance of nurses in patient education—particularly in training for self-injection and promoting a healthy lifestyle and mental well-being. Their involvement was strongly associated with higher patient satisfaction and was a significant contributor to the overall effectiveness of care in CIA management. Patients, among all other respondents, demonstrated the highest level of confidence in nurses’ competencies to attend to their needs. The only exception was the issue of dealing with treatment adverse reactions, where rheumatology doctors were the most preferred medical professionals to consult.

The data presented can serve as a foundation for future studies focused on developing training programs for nurses in rheumatology settings. Further research is needed to better understand how nurse-led self-management training impacts the quality of care for patients with chronic inflammatory arthritis. Additionally, investigating the long-term outcomes of these interventions could yield valuable insights into patient adherence and overall disease management.

## Figures and Tables

**Table 1:** Opinions on nurses’ competency to train patients in self-injection and healthy lifestyles.

Do You Think That Nurses Have the Competency to…	Patients n = 219	Nurses n = 34	Doctors n = 29	*p*-Value
Train patients to administer self-injection				
○ Yes	193 (88.1%) ^a^	27 (79.4%) ^a,b^	19 (65.5%) ^b^	0.002
○ Rather yes	13 (5.9%) ^a^	6 (17.6%) ^a,b^	9 (31.0%) ^b^	
○ Neutral	11 (5.0%) ^a^	1 (2.9%) ^a^	1 (3.4%) ^a^	
○ Rather no	0 (0.0%) ^a^	0 (0.0%) ^a^	0 (0.0%) ^a^	
○ No	2 (0.9%) ^a^	0 (0.0%) ^a^	0 (0.0%) ^a^	
Educate patients to engage in physical activities				
○ Yes	150 (68.5%)	19 (55.9%)	15 (51.7%)	
○ Rather yes	36 (16.4%)	9 (26.5%)	9 (31.0%)	
○ Neutral	23 (10.5%)	1 (2.9%)	4 (13.8%)	0.086
○ Rather no	7 (3.2%)	4 (11.8%)	1 (3.4%)	
○ No	3 (1.4%)	1 (2.9%)	0 (0.0%)	
Offer guidance on nutrition, harmful habits and other health-related issue				
○ Yes	149 (68.0%) ^a^	22 (64.7%) ^a,b^	13 (44.8%) ^b^	
○ Rather yes	36 (16.4%) ^a^	9 (26.5%) ^a,b^	14 (48.3%) ^b^	
○ Neutral	25 (11.4%) ^a^	0 (0.0%) ^a^	1 (3.4%) ^a^	0.002
○ Rather no	6 (2.7%) ^a^	1 (2.9%) ^a^	1 (3.4%) ^a^	
○ No	3 (1.4%) ^a^	2 (5.9%) ^a^	0 (0.0%) ^a^	
Provide psychological and emotional support				
○ Yes	179 (82.7%)	23 (67.6%)	19 (65.5%)	
○ Rather yes	24 (11.0%)	7 (20.6%)	6 (20.7%)	
○ Neutral	11 (5.0%)	2 (5.9%)	4 (13.8%)	0.122
○ Rather no	2 (0.9%)	0 (0.0%)	0 (0.0%)	
○ No	3 (1.4%)	2 (5.9%)	0 (0.0%)	

Cells marked with different superscript letters denote significant differences between the groups at the 0.05 level.

## Data Availability

Access to the data can be obtained from the corresponding author upon reasonable request.

## Abbreviations

The following abbreviations are used in this manuscript:

CIA	Chronic inflammatory arthritis
RA	Rheumatoid arthritis
AS	Ankylosing spondylitis
PsA	Psoriatic arthritis
bDMARDs	Biologic disease-modifying antirheumatic drugs
RLC	Rheumatologist-led care
NLC	Nurse-led care
